# High Level of Plasma Matrix Metalloproteinase-11 Is Associated with Clinicopathological Characteristics in Patients with Oral Squamous Cell Carcinoma

**DOI:** 10.1371/journal.pone.0113129

**Published:** 2014-11-25

**Authors:** Chung-Han Hsin, Mu-Kuan Chen, Chih-Hsin Tang, Huang-Pin Lin, Ming-Yung Chou, Chiao-Wen Lin, Shun-Fa Yang

**Affiliations:** 1 School of Medicine, Chung Shan Medical University, Taichung, Taiwan; 2 Department of Otolaryngology, Chung Shan Medical University Hospital, Taichung, Taiwan; 3 Institute of Medicine, Chung Shan Medical University, Taichung, Taiwan; 4 Department of Otorhinolaryngology-Head and Neck Surgery, Changhua Christian Hospital, Changhua, Taiwan; 5 Graduate Institute of Basic Medical Science, China Medical University, Taichung, Taiwan; 6 Department of Biotechnology, College of Health Science, Asia University, Taichung, Taiwan; 7 Institute of Oral Sciences, Chung Shan Medical University, Taichung, Taiwan; 8 Department of Dentistry, Chung Shan Medical University Hospital, Taichung, Taiwan; 9 Department of Medical Research, Chung Shan Medical University Hospital, Taichung, Taiwan; University of Patras, Greece

## Abstract

**Background:**

Matrix metalloproteinase-11 (MMP-11) is reported to be overexpressed in several cancers and may contribute to tumorigenesis. The current study investigated the association between the clinicopathological characteristics and plasma level of MMP-11 in oral squamous cell carcinoma (OSCC) patients.

**Methodology and Principal Findings:**

The plasma MMP-11 concentration was determined by ELISA on 330 male OSCC patients. In addition, the metastatic effects of the MMP-11 knockdown on the oral cancer cells were investigated by cell migration assay. Our results showed that the plasma MMP-11 levels were significantly higher in patients with advanced T status (p = 0.001), lymph node metastasis (p = 0.006) and higher TNM stages (p<0.001). Moreover, treatment with the MMP-11 shRNA exerted an inhibitory effect on migration in SCC9 oral cancer cells.

**Conclusion:**

Our study showed that plasma level of MMP-11 may be useful for assessment of the disease progression, especially lymph node metastasis, in patients with OSCC.

## Introduction

Oral squamous cell carcinoma (OSCC) is the most common malignancy of the head and neck region, accounting for 2–4% of all cancer cases in Western countries and even more than 10% in some areas of the Asia [1], [2]. Currently, the mainstay treatment for OSCC includes extensive surgical excision with or without adjuvant radiotherapy and/or chemotherapy [3]. However, even with these aggressive interventions, the 5-year relative survival rate remains unfavorable in a considerable portion of OSCC patients because invasion of the neighboring tissues and metastasis to the neck lymph nodes by cancer cells are common [4]. Therefore, identifying new biomarkers that can predict the risk of OSCC progression, especially local invasion and lymph node metastasis, is needed to improve the control of this deadly form of cancer.

Tumor invasion and metastasis involve a series of complex processes, including cell adhesion, migration, invasion, angiogenesis, and anchorage-independent growth [5]. Among them, degradation of the extracellular matrix (ECM) and access of blood vessels and lymphatics for cancer cells is one of the key processes. Matrix metalloproteinases (MMPs) are zinc-dependent endopeptidases that efficiently degrade the components of the ECM and basement membranes, and increased production of these enzymes is observed in almost all human cancers and has been associated with the invasive and/or metastatic phenotypes in many tumors [6], [7]. Matrix metalloproteinase-11 (MMP-11) is also known as Stromelysin-3, and was initially identified in invasive breast carcinomas. It is associated with intense tissue remodeling during tissue involution, embryogenesis and wound healing in normal physiologic conditions [8]. Unlike most MMP family members, MMP-11 does not cleave major components of the ECM. Furthermore, whereas most MMPs are secreted as proenzymes that need extracellular activation, MMP-11 is processed intracellularly and secreted as an active enzyme. This suggests that this endopeptidase may have a unique role in tumor development and progression [9], [10]. Increased expression of MMP-11 has been observed in most invasive human carcinomas, including breast, lung, colorectal and ovarian carcinomas, and high levels of its mRNA was reportedly correlated with aggressive phenotypes and poor clinical outcome [11]–[13].

The clinicopathological significance of MMP-11 has also been demonstrated by immunohistochemical staining on tumor specimens of OSCC patients [14]–[16]. Soni et al found that, in surgically resected tissue specimens or biopsy samples of OSCC patients, expression of MMP-11 was significantly associated with the involvement of neck lymph node [15]. Another study investigated the expression levels of MMP-11 and relevant proangiogenic factors in OSCC and precancerous lesions, and found that concomitant expression of MMP-11 and proangiogenic factors was important predictor for progression from precancerous stage to malignancy [16]. These findings, however, were mainly based on immunohistochemical analyses on surgical tumor specimens or biopsied samples. The level of plasma MMP-11 in patients with OSCC is still not clear. The current study investigated the association between the clinicopathological characteristics and plasma level of this endopeptidase in OSCC patients.

## Materials and Methods

### Subjects and Specimen Collection

We recruited 330 OSCC male patients (mean age of 54.99±10.88 years) at Chung Shan Medical University Hospital in Taichung and Changhua Christian Hospital in Changhua, Taiwan between 2008 and 2012. Medical information of the OSCC patients, including TNM clinical staging, primary tumor size, lymph node involvement, and histological grade, was obtained from their medical records. Patients with OSCC were clinically staged at the time of diagnosis according to the TNM staging system of American Joint Committee on Cancer (AJCC) Staging Manual, seventh edition. The whole blood samples were collected from OSCC patients and placed in tubes containing ethylenediaminetetraacetic acid (EDTA). After immediate centrifugation at 3000 rpm, the supernatants were stored at –80°C. Before conducting this study, approval from the Institutional Review Board of Chung Shan Medical University Hospital and informed written consent to participate in the study were obtained from each individual.

### Quantitative analysis of plasma MMP-11 level

The plasma MMP-11 concentration was determined quantitatively by the enzyme-linked immunosorbent assay (ELISA) according to the manufacturer’s specification (Human MMP-11 Immunoassay) (sE90224Hu); (USCN Life Science, Wuhan, China). One hundred microliters of plasma samples (100 fold dilution), standard control samples and internal quality controls were dispensed into the microtitre plates coated with a monoclonal antibody against MMP-11 and incubated for 2 hours at room temperature on a horizontal orbital shaker at 200 rpm. After washing the plates to remove unbound protein, 100 µL of detection reagent A solution was added and incubated for 1 hour at room temperature on a shaker. After added 100 µL of detection reagent B, the plates were washed, and then further incubated with 90 µL of substrate solution for 20 minutes at room temperature protected from light. The color development was stopped after 30 min by adding 50 µL of stop solution and the absorbance was measured at 450 nm by a spectrophotometer microplate reader. The MMP-11 levels were quantified with a calibration curve using human MMP-11 as a standard.

### Cell culture

The SCC9, SCC25 and HSC3 human oral cancer cell lines were purchased from ATCC (ATCC: American Type Culture Collection, Manassas, VA, USA). The SAS human oral cancer cell lines were purchased from JCRB (JCRB: Japanese Collection of Research Bioresources, Osaka, Japan). TW2.6 cells were kind gifts of Dr. Cheng-Chia Yu at Chung Shan Medical University (Taichung, Taiwan) [17]. HSC3 cells were cultured in Dulbecco’s modified Eagle’s medium (Life Technologies, Grand Island, NY, USA). SCC9, SCC25, SAS and TW2.6 cells were cultured in Dulbecco’s modified Eagle’s medium supplemented with an equal volume of a nutrient mixture, F-12 Ham’s medium (Life Technologies, Grand Island, NY, USA). All cell cultures were maintained at 37°C in a humidified atmosphere of 5% CO_2_.

### Establishment of short hairpin (sh)RNA-MMP-11 knockdown in oral cancer Cells

Lentiviral constructs of shRNA were purchased from the National RNA Interference Core Facility (Institute of Molecular Biology, Academia Sinica, Taipei, Taiwan). A construct (TRCN0000050713) of shRNA targeting MMP-11 and shLuc (as non-targeting shRNA control) were used. For lentivirus production, the supernatant of 293T cells was harvested 48 hr after transfection with shRNA vectors. Targeted cells were then incubated with lentiviruses and 8 mg/ml polybrene (Sigma–Aldrich) for 24 hr. MMP-11 knockdown in SCC9 oral cancer cells was confirmed by Western blot analysis and qRT-PCR assay.

### Western Blot Analysis

Cellular lysates were prepared by suspending 2×10^6^/10 cm dish in 200 µL of RIPA buffer containing protease inhibitors cocktail. Cell lysates were subjected to a centrifugation of 10,000 rpm for 10 min at 4°C, and the insoluble pellet was discarded. The protein concentration of cell lysates was determined by Bradford assay. The 20 µg samples of cell lysates was separated by SDS-PAGE on 10% polyacrylamide gels and transferred onto a nitrocellulose membrane using the Mini-Protean Tetra Electrophoresis System as described previously [18]. The blot was subsequently incubated with 5% non-fat milk in Tris-buffered saline (20 mM Tris, 137 mM NaCl, pH 7.6) for 1 h to block non-specific binding and then overnight with polyclonal antibodies against MMP-11 (Santa Cruz, CA, USA; Cat# sc-8836-R; 1:1000 dilutions) and β-actin (Novus Biologicals, Co, USA; Cat# NB600-501; 1:5000 dilutions). Moreover, HeLa whole cell lysate is recommended for detection of MMP-11 as the positive control to ensure the specificity of the bands. Blots were then incubated with a horseradish peroxidase anti-rabbit IgG for 1 h. Afterwards, signal was detected by using enhanced chemiluminescence (ECL) commercial kit (Amersham Biosciences).

### RNA Isolation and TaqMan Quantitative Real-time PCR

Total RNA was isolated from 1×10^6^ HSC3, SAS, SCC9, SCC25 and TW2.6 cells using Trizol (Life Technologies, Grand Island, NY) according to the manufacturer’s instructions. The quality of the isolated RNA was detected by a measurement of OD260/OD 280 ratio. Our RNA has an A260/A280 of 2.0. Total RNA (2 µg) was reverse transcribed into cDNA by SuperScript III First-Strand Synthesis Supermix (Invitrogen, Carlsbad, CA). Quantitative real-time PCR analysis was carried out using Taqman one-step PCR Master Mix (Applied Biosystems). 100 ng of total cDNA was added per 25 µl reaction with MMP-11 or GAPDH primers and Taqman probes. The MMP-11 and GAPDH primers and probes were designed using commercial software (ABI PRISM Sequence Detection System; Applied Biosystems). Quantitative real-time PCR assays were carried out in triplicate on a StepOnePlus sequence detection system. The threshold was set above the non-template control background and within the linear phase of target gene amplification to calculate the cycle number at which the transcript was detected.

### Cell Migration Assays

After a treatment with the shLuc (as non-targeting shRNA control) and MMP-11 shRNA for 24 h, SCC9 cells were harvested and seeded to Boyden chamber (Neuro Probe, Cabin John, MD, USA) at 10^4^ cell/well in serum free medium and then incubated for 24 h at 37°C. The invaded cells were fixed with 100% methanol and stained with 5% Giemsa. Cell numbers were counted under a light microscope.

### Statistical Analysis

The data of demography were presented by number (%) and mean ± standard deviation (SD). The significances of differences between means were calculated by Student's t-test. In addition, gender, smoking status, drinking status and betel nuts chewing were analyzed by *x^2^* test. p<0.05 was considered as statistically significant. Analyses were performed using SPSS 16.0 statistical software (SPSS Inc., Chicago, IL, USA).

## Results

### Patient Characteristics

Three hundred and thirty patients with OSCC were included in the current analyses. Table 1 presents the demographic data, which showed that 89.4% patients with OSCC were smokers, 58.2% patients with OSCC were alcohol consumers, and 78.2% patients with OSCC chewed betel nuts. Furthermore, among the 330 cases, tumors were located in the buccal mucosa (n = 120), tongue (n = 98), gingiva (n = 39), and others (n = 63). TNM status and types of tumor cell differentiation of OSCC patients are also shown in Table 1.

**Table 1:** Demographic characteristics and clinical features of OSCC patients.

Variables	OSCC (n = 330)
**Age** (years)	54.99±10.88
**Smoking status**	
No	35 (10.6%)
Yes	295 (89.4%)
**Drinking status**	
No	138 (41.8%)
Yes	192 (58.2%)
**Betel nuts chewing**	
No	72 (21.8%)
Yes	258 (78.2%)
**Cancer location**	
Buccal mucosa	120 (36.4%)
Tongue	98 (29.7%)
Gingiva	49 (14.8%)
Others	63 (19.1%)
**Stage**	
I	72 (21.8%)
II	59 (17.9%)
III	36 (10.9%)
IV	163 (49.4%)
**Tumor T status**	
T1	92 (27.9%)
T2	97 (29.4%)
T3	32 (9.7%)
T4	109 (33.0%)
**Lymph node status**	
N0	201 (60.9%)
N1	43 (13.0%)
N2	83 (25.2%)
N3	3 (0.9%)
**Metastasis**	
M0	328 (99.4%)
M1	2 (0.6%)
**Cell differentiation**	
Well differentiated	35 (10.6%)
Moderately or poorly differentiated	295 (89.4%)

### Correlation between plasma MMP-11 levels and clinicopathological characteristics of patients

**Figure 1:**
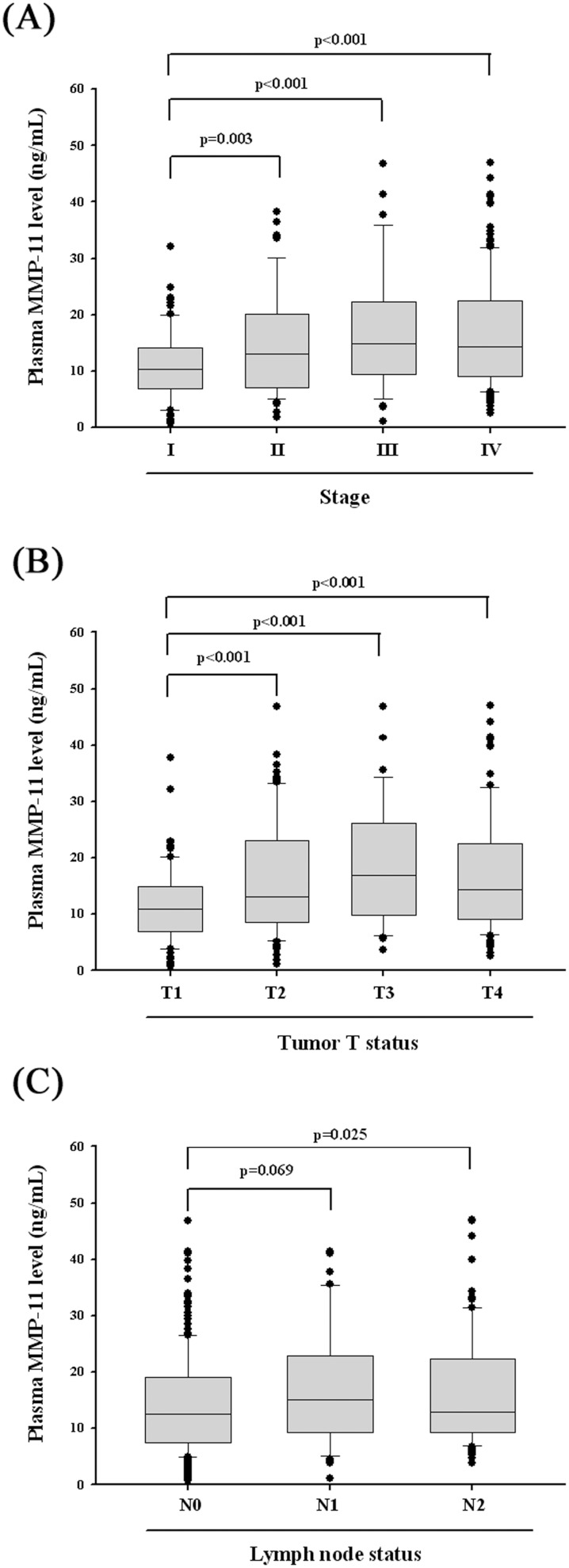
ELISA-determined plasma MMP-11 level of OSCC patients at various clinical features. (A) MMP-11 levels were compared according to stage and results showed that MMP-11 levels were significantly higher in tumor stage II, III and IV when compared with stage I patients. (B) MMP-11 levels were compared according to tumor T status and results showed that MMP-11 levels were significantly higher in advanced tumor extent when compared with T1 patients. (C) MMP-11 levels were compared according to N status and results showed that MMP-11 levels were significantly higher in N2, but not in N1 when compared with patients without nodal metastasis.

The relationship between plasma MMP-11 levels and various clinicopathological parameters of OSCC patient is summarized in Table 2. Plasma levels of MMP-11 protein were not correlated with age, sex, smoking, drinking, betel nuts chewing, cancer location, distant metastasis and cell differentiation. However, MMP-11 protein levels were significantly higher in patients suffering with advanced T status (T3+T4; p = 0.001), lymph node metastasis (N1+N2+N3; p = 0.006) and higher TNM stages (stage III + stage IV; p<0.001). Detailed comparisons of plasma MMP-11 between OSCC patients with different disease severities are further illustrated in Figure 1. The levels of plasma MMP-11 were significantly higher in patients with TNM stages II (15.19±9.30 ng/mL), III (17.34±11.21 ng/mL) and IV (16.99±10.04 ng/mL) when compared with stage I (11.02±6.23 ng/mL) patients (Figure 1A). The levels of MMP-11 were significantly higher in patients with advanced tumor extent (T2: 16.10±9.74 ng/mL, T3: 18.58±10.82 ng/mL and T4: 17.16±10.39 ng/mL) when compared with T1 (11.48±6.56 ng/mL) patients (Figure 1B). As to N stage, the levels of MMP-11 were significantly higher in patients with N2 (16.96±9.93 ng/mL), but not N1 (17.12±10.05 ng/mL), when compared with patients without nodal metastasis (14.25±8.98 ng/mL) (Figure 1C).

**Table 2:** Correlation between plasma levels of MMP-11 and clinicopathological parameters in 330 OSCC patients.

	No. of case (%)	MMP-11 level	p value
Variables	n = 330	Mean ± S.D. (ng/mL)	
**Age** (years)			
<55	166 (50.3%)	15.69±10.12	0.592
≥55	164 (49.7%)	15.12±9.10	
**Smoking status**			
No	35 (10.6%)	14.08±9.44	0.388
Yes	295 (89.4%)	15.56±9.64	
**Drinking status**			
No	138 (41.8%)	14.85±9.45	0.377
Yes	192 (58.2%)	15.80±9.74	
**Betel nuts chewing**			
No	72 (21.8%)	14.36±9.51	0.297
Yes	258 (78.2%)	15.70±9.65	
**Cancer location**			
Buccal mucosa	120 (36.4%)	14.17±9.00	0.195
Tongue	98 (29.7%)	15.24±9.61	
Gingiva	49 (14.8%)	16.60±10.88	
Others	63 (19.1%)	17.10±9.61	
**Stage**			
I+II	131 (39.7%)	12.90±8.01	**<0.001** *
III+IV	199 (60.3%)	17.06±10.23	
**Tumor T status**			
T1+T2	189 (57.3%)	13.85±8.64	**0.001** *
T3+T4	141 (42.7%)	17.48±10.47	
**Lymph node status**			
N0	201 (60.9%)	14.25±8.98	**0.006** *
N1+N2+N3	129 (39.1%)	17.21±10.32	
**Metastasis**			
M0	328 (99.4%)	15.31±9.56	0.183
M1	2 (0.6%)	30.86±6.65	
**Cell differentiation**			
Well differentiated	35 (10.6%)	15.36±9.38	0.975
Moderately or poorly differentiated	295 (89.4%)	15.41±9.66	

*p<0.05.

### MMP-11 shRNA reduced the migration of oral cancer cell lines

**Figure 2:**
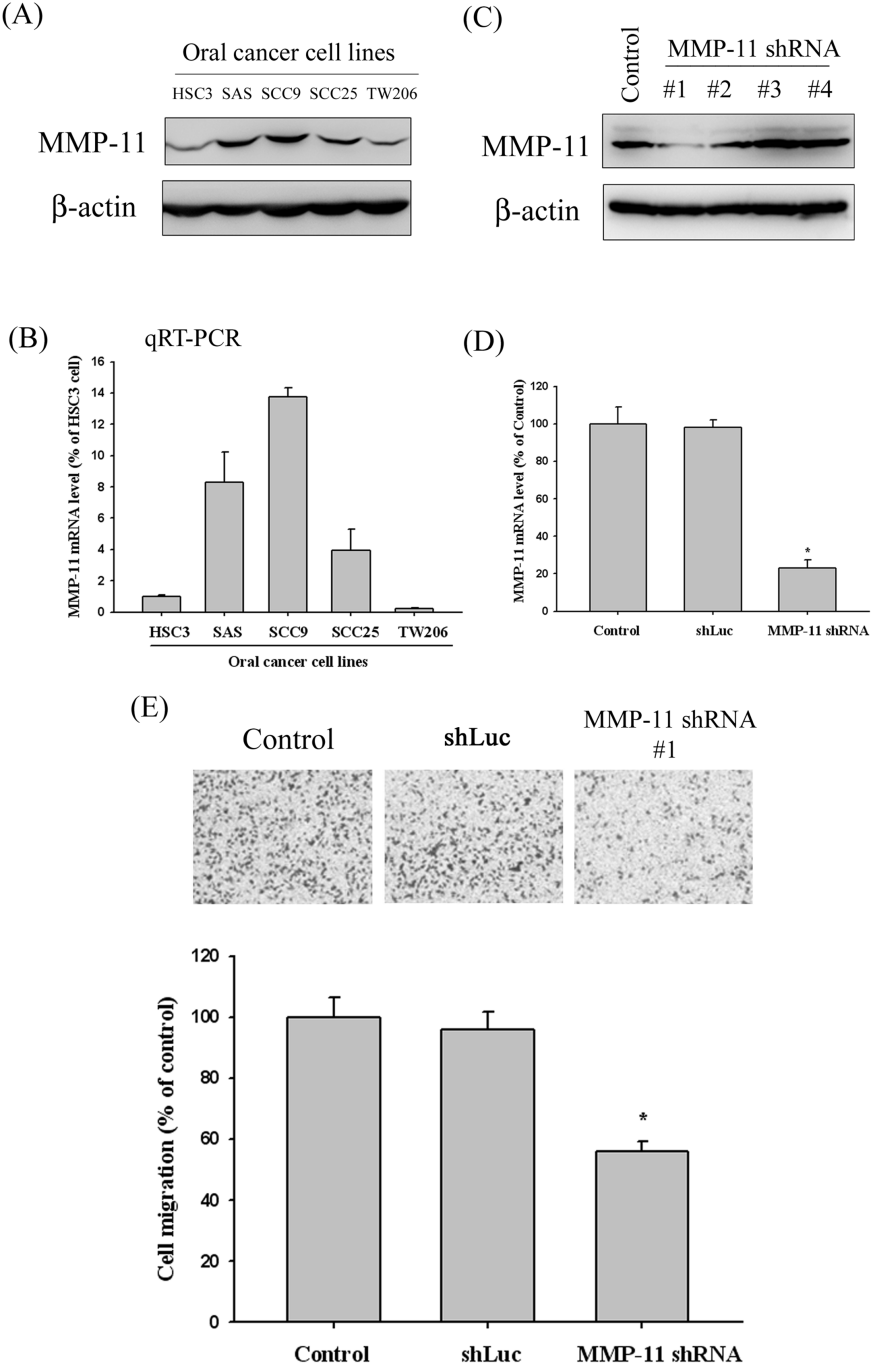
MMP-11 expression in oral cancer cell lines and the MMP-11 knockdown in SCC9 cells reduce cell migration. (A) Western blot analysis showing the expression of MMP-11 protein in five oral cancer cell lines. (B) MMP-11 mRNA levels in five oral cancer cell lines were determined by quantitative real-time PCR using β-actin as an endogenous control. Results are shown as mean ± SD of three independent experiments. (C) Endogenous MMP-11 expression is silenced by infection with 4 different shRNA constructs (#1 and #2, #3 and #4) for MMP-11 in SCC9 oral cancer cells. Knockdown efficiency was analyzed by Western blotting. Results showed an approximately 80% reduction of #1 shRNA of MMP-11. *p<0.05. (D) MMP-11 Knockdown efficiency in SCC9 cell was confirmed by quantitative real-time PCR. (E) Detection of cell migration ability by infection with shLuc or shRNA of MMP-11 in SCC9 cell. Results shown that MMP-11 shRNA significantly reduced the migration of SCC9 cells. *p<0.05.

Since we found that expression of MMP-11 was significantly correlated with the presence of lymph node metastasis, the effects of the MMP-11 knockdown on the oral cancer cell line were investigated by cell migration assay. To select oral cancer cell lines suitable for MMP-11 knockdown experiments, we examined the MMP-11 protein and mRNA in five oral cancer cell lines (HSC3, SAS, SCC9, SCC25 and TW2.6) by Western blot and qRT-PCR analysis. Our data showed that MMP-11 expression was significantly higher in SCC9 and SAS, as compared to the one in HSC3, SCC25 and TW2.6 oral cancer cell (Fig. 2A). The qRT-PCR results are consistent with the Western blotting results (Fig. 2B). Thus, we used a Lentiviral-shRNA (shRNA) approach to inhibit endogenous MMP-11 expression in the SCC9 cell lines. We used 4 different shRNA constructs (#1 and #2, #3 and #4) for MMP-11. Compared with the shLuc control (non-targeting control), results from Western blotting showed an approximately 80% reduction of MMP-11 expression in the #1 MMP-11 shRNA constructs (Fig. 2C). This effect of MMP-11 reduction was also confirmed by qRT-PCR (Fig. 2D). Using the cell migration assay with a Boyden chamber, it was shown that MMP-11 shRNA significantly reduced the migration of SCC9 cells, with only 56% remaining after treatment with MMP-11 shRNA (Fig. 2E).

## Discussion

MMPs are considered important in the carcinogenic process due to their proteolytic ability [19], [20], although later studies have demonstrated that they have more complex role in tumorigenesis [21]–[25]. Almost all members of the MMP-family have been implicated in the genesis of various human cancers, including OSCCs [7], [26]. In the current study, we for the first time demonstrated the biomarker potential of MMP-11 plasma level for predicting clinicopathological characteristics in OSCC patients.

For head and neck cancers, MMP-1, MMP-2, MMP-9 and membrane type-1 MMP have been found overexpressed in the tumor tissues and been associated with cancer progression [26], [27]. MMP-11 on the other hand, has been less investigated in this regard. Soni et al performed immunohistochemical analysis of MMP-11 expression in tumor specimens of 177 OSCC patients and found positive expression in 123 (70%) of these [15]. Their data also demonstrated that MMP-11 positivity was associated with lymph node involvement, although Kaplan-Meier analysis failed to reveal association between MMP-11 expression and survival of OSCC patients. Another study by Arora et al, which investigated the expression of MMP-11 and relevant proangiogenic factors in 220 OSCC and 90 precancerous lesions, showed that concomitant expression of MMP-11 and proangiogenic factors was an important predictor for progression from precancerous stage to malignancy [16]. Although these data can serve as references for the application of MMP-11 as a tumor marker, the specimens in these studies were obtained from surgical tumors or biopsied samples.

The advantage of plasma tumor markers, on the other hand, is that plasma can be easily obtained before treatments and during any time of the patient’s follow-up without the necessity of invasive excising of tissue. Previous studies of head and neck cancers have discovered that MMP-9 plasma concentrations correlate with disease stages of the cancer and MMP-8 plasma levels correlate with T-status, N-status and disease staging [28], [29]. The subject of these studies, however, was mainly pharyngeal and laryngeal cancers instead of OSCCs. A recent study of 148 OSCC patients found that elevated plasma Decoy receptor 3, a member of tumor necrosis factor receptors, was associated with nodal metastasis and worse prognosis [30].

Our investigation on MMP-11 in 330 male OSCC patients demonstrated that the plasma levels of MMP-11 protein correlated with primary tumor extent, nodal metastasis (except N1) and TNM staging of the disease (Figure 1). Moreover, to test the biological effects of reduced expression of MMP-11 protein on OSCC cell, migration assay of oral cancer cell line with knockdown MMP-11 was conducted, and demonstrated treatment with the MMP-11 shRNA exerted an inhibitory effect on migration in SCC9 oral cancer cells (Fig. 2E). These data suggested that MMP-11 may play an important role in the carcinogenesis of OSCC, and the detection of MMP-11 protein in the plasma might serve as tumor marker predicting the likelihood for patients without OSCC.

It is clinically invaluable to have biomarkers which can provide information regarding the likelihood of lymph node metastasis. This is particularly important in the context of OSCCs because there is clear evidence that in patients who are clinically staged with a N0 status disease, often occult neck node metastases are already present [31], [32]. Finding reliable tumor markers for OSCC patients would help not only for estimation of the prognosis, but also for the prediction of tumor behavior and adequate therapy planning. Consequently, our findings that elevated plasma levels of MMP-11 correlate with nodal metastasis should have an important implication in the treatment of OSCCs.

It should be mentioned, however, that although mean MMP-11 level in plasma of N1 patients was higher to those of N2 patients, it did not show significant difference with the respective of patients with lymph node metastasis (Fig. 1C). The reasons may include: first, the patient number of N1 was smaller than N2 (45 vs 85) and, second, the standard deviation for MMP-11 level was larger for N1 group than N2 group (17.12+10.05 ng/mL vs 16.96+9.93 ng/mL). Because our data failed to show a significant difference of MMP-11 level in plasma between N1 and N0 diseases, plasma MMP-11 probably cannot separate reliably between N0 and N1 diseases, although enrollment of more patients in the future may find a significant difference between the two.

Except for oral cancer, overexpression of plasma MMP-11 has also been demonstrated as a useful marker for diagnosis, prognosis and treatment in other cancers. High plasma levels of MMP-11 were found to be associated with lymph node metastasis and prognosis in patients with gastric cancer, especially advanced carcinoma [33]. A recent study also showed that MMP-11 could be as a promising biomarker for breast cancer and a suitable target for cancer immunotherapy strategies [34]. Together with our results, plasma concentration of MMP-11 protein appears to be a promising candidate biomarker for various human cancers and deserves clinical verification by further studies.

To the best of our knowledge, this is the first study to examine the association between plasma MMP-11 level and clinicopathological characteristics for OSCC patients, with an attempt to explore the application of this molecule as a tumor marker. Limitations, however, do exist in our investigation and should be highlighted. First, the information on alcohol, betel nut, and tobacco use is dichotomized into “ever-user” versus “never-user”. As a result, more detailed analysis based on amount and length of tobacco consumption was not able to be performed. Second, inability to perform Kaplan-Meier survival analysis impedes the investigation about association between plasma MMP-11 and disease prognosis. Third, regarding the migration analyses in vitro, we have not checked if MMP-11 knock-down affected proliferation or apoptotic rate of the SCC9 cell. In future studies, we will include control subjects and take more OSCC risk factors into account in the analysis to adequately validate these findings. Furthermore, with a long enough follow-up period for these OSCC patients, we will be able to perform a Kaplan-Meier analysis to look at the relationship of plasma MMP-11 and disease prognosis.

In conclusion, our study indicated that a substantial increase in the plasma level of MMP-11 by performing ELISA assay is useful for assessment of the disease progression, especially lymph node metastasis, in patients with OSCC. Furthermore, in vitro study also showed that MMP-11 knockdown had an inhibitory effect on migration in SCC9 oral cancer cell. As a secreted protein, MMP-11 may play an important role in carcinogenesis and find clinical applications as a biomarker for disease diagnosis and therapy planning for human OSCCs.
